# Expression, Purification, and Monitoring of Conformational Changes of hCB2 TMH67H8 in Different Membrane-Mimetic Lipid Mixtures Using Circular Dichroism and NMR Techniques

**DOI:** 10.3390/membranes7010010

**Published:** 2017-02-17

**Authors:** Elvis K. Tiburu, Jianqin Zhuang, Heidimarie N. A. Fleischer, Patrick K. Arthur, Gordon A. Awandare

**Affiliations:** 1 Department of Biomedical Engineering, University of Ghana, P.O. Box LG 25, Legon, Accra, Ghana; 369hmnaf@gmail.com; 2 Department of Chemistry, College of Staten Island, New York, NY 10314, USA; jianqinzhuang@gmail.com; 3 Department of Biochemistry, Cell and Molecular Biology; University of Ghana, P.O. Box LG 25, Legon, Accra, Ghana; parthur14@gmail.com (P.K.A.); gawandare@ug.edu.gh (G.A.A.); 4 West Africa Center for Cell Biology of Infectious Pathogens (WACCBIP), Accra, Ghana

**Keywords:** selective and uniformly labeling, insect cells, bicelles, NMR

## Abstract

This work was intended to develop self-assembly lipids for incorporating G-protein coupled receptors (GPCRs) in order to improve the success rate for nuclear magnetic resonance spectroscopy (NMR) structural elucidation. We hereby report the expression and purification of uniformly ^15^N-labeled human cannabinoid receptor-2 domain in insect cell media. The domain was refolded by screening several membrane mimetic environments. Different *q* ratios of isotropic bicelles were screened for solubilizing transmembrane helix 6, 7 and 8 (TMH67H8). As the concentration of dimyristoylphosphocholine (DMPC) was increased such that the *q* ratio was between 0.16 and 0.42, there was less crowding in the cross peaks with increasing *q* ratio. In bicelles of *q* = 0.42, the maximum number of cross peaks were obtained and the cross peaks were uniformly dispersed. The receptor domain in bicelles beyond *q* = 0.42 resulted in peak crowding. These studies demonstrate that GPCRs folding especially in bicelles is protein-specific and requires the right mix of the longer chain and shorter chain lipids to provide the right environment for proper folding. These findings will allow further development of novel membrane mimetics to provide greater diversity of lipid mixtures than those currently being employed for GPCR stability and folding, which are critical for both X-ray and NMR studies of GPCRs.

## 1. Introduction

The G-protein coupled receptor (GPCR) superfamily constitutes a large and diverse family of proteins in eukaryotic membranes and are targets of most drugs [1,2,3]. They comprise a bundle of seven transmembrane α helices connected by intracellular and extracellular loops [4]. Tremendous challenges in their expression and purification, coupled with optimization of appropriate membrane-mimetic systems for reconstituting GPCRs, have isolated the GPCR family as the largest subset of transmembrane proteins yet to be fully characterized by biophysical techniques [5]. This has hampered interpretation of biochemical findings, and thus limits the development of rational drug discovery among therapeutically important GPCRs. Among the structural biology techniques applied to GPCR characterization, X-ray crystallography has enjoyed much success in providing high-resolution structures of isolated GPCRs in complex with selected ligands reconstituted in detergent micelles [6,7]. The first crystal structure to be determined was that of rhodopsin, which was stabilized by a covalently bound ligand that held the receptor in an inactive state [7]. Efforts to explain the complex behavior of GPCRs and their ability to recognize a wide range of ligands led to other crystal structures of engineered receptors, among which are the human β_2_-adrenergic receptor (hβ_2_AR), turkey β_1_AR, as well as human D3 dopamine receptors (hD3Rs), also in detergent micelles [8,9,10]. Thus, the structural characterization of GPCRs and understanding their mechanisms of action have recently been elevated in importance as a means to improve the success of drug discovery [1,11].

The major issues in studying GPCRs with NMR are the challenges in controlling the conformational plasticity and thermal stability (T_m_) of the receptor in detergent micelles to ensure the system is within the limits of NMR experiments [5,12,13]. The most widely used environments to produce properly folded membrane proteins for NMR studies are through the reconstitution in membrane-mimetic environments, such as organic solvents [14,15,16] and detergent micelles [17,18,19]. Another technique to study membrane proteins in lipid bilayers involves the utilization of a combination of a short-chain detergent, such as dihexanoylphosphocholine (DHPC), and a longer chain lipid, such as dimyristoylphosphocholine (DMPC), at the appropriate concentrations to yield discoidal membranes called “bicelles” [18,20,21,22,23,24].

Recently, monodispersed nanodisc environments have also been adopted to stabilize the seven-transmembrane (7-TM) bundle to maintain the conformational dynamics and functionality of chemokine receptor type 5 (CXCR5) and hβ_2_AR receptors [5,25]. Structural studies of pSRII, a homolog protein of GPCRs, also suggest that choosing the best membrane mimetic is key to the preparation of homogenous sample for obtaining a good NMR spectrum [26]. Organic solvents such as trifluoroethanol (TFE) and 1,1,1,3,3,3-hexafluoro-2-propanol (HFIP) have also been applied to study fragments of GPCRs using NMR techniques [14,27,28,29]. All these results further support that NMR is a very important tool in the structural determination of membrane proteins for drug discovery, as long as the right microenvironment is identified.

The human cannabinoid receptor 2 (hCB2), a member of the class A type GPCR, is a potential drug target and yet, to date, direct structural information regarding its function is very limited [5,7,30]. As in other GPCRs, hCB2 is defined by iconic seven-transmembrane helix (7-TMH) bundle connected by intracellular and extracellular loops, with TMH1–5 comprising one domain and TMH6,7 another domain [31]. From occluded surface area calculations, most ligands targeting type A GPCRs make contact with TMH6,7, with sporadic selection of domains from TMH1–5 [32]. Other ligands target extracellular portions of TMH1–5, where the reorientation of the positions of these helices in detergent micelles appears to redefine the location of the ligand-binding pocket. The inability to define accurately the ligand-binding site is due to GPCRs’ conformational plasticity arising from enhanced dynamics within detergent micelles [33,34,35]. Therefore, the membrane environment has a significant role to play in the activity of GPCRs such as the hCB2 receptor. In this current work, efforts were devoted to screening a library of membrane-mimetic environments for solubilizing hCB2 TMH67H8 receptor domain for the purpose of structural studies. First, through modifications to commercially available insect cell growth media, selectively ^15^N-labeled TMH67H8 was produced in which all the leucine (Leu) residues were replaced by their isotopic variants to screen various membrane mimetics for the NMR studies. Then, the same variant was expressed with our modified insect cell media to produce ^15^N and ^13^C double-labeled TMH67H8 for further analysis of the conformation of the receptor domain in the best membrane environment.

TMH67H8 virus of hCB2 was amplified using the titerless infected-cells preservation and scale-up method for producing milligram quantities of the receptor domain [36]. The incorporation of the hCB2 receptor domain into selected detergent micelles and isotropic bicelles were conducted utilizing a modified approach. NMR experiments were then conducted utilizing ^1^H-^15^N transverse relaxation-optimized spectroscopy (TROSY) pulse sequence on both selectively and uniformly labeled hCB2 receptor domains to determine the best conditions for folding and the implication of the lipid environment on the conformation of TMH67H8. Among the membrane mimetics screened, the bicelle environment provided remarkable variations in the ^1^H-^15^N TROSY spectra through changes in the DMPC/DHPC lipid ratio, indicative of a properly folded TMH67H8.

## 2. Experimental Procedures

### 2.1. Materials

*n*-Dodecyl-β-d-maltopyranoside (DDM) was purchased from Affymetrix (Santa Clara, CA, USA). *n*-Dodecylphosphocholine (DPC), TFE, and HFIP were obtained from Cambridge Isotopes (Andover, MA, USA), and all lipids were obtained from Avanti Polar Lipids (Alabaster, AL, USA).

### 2.2. hCB2 TMH67H6 Expression and Purification

*Recombinant DNA Construction:* The nucleotides corresponding to the human cannabinoid receptor-2 TMH6,7H8 was designed such that there was a *Bam*HI and *Nco*l sites at the N-terminus and *Eco*RI at the C-terminus. The N-terminus was flag-tagged, whereas the six histidines were attached at the C-terminus to facilitate purification using affinity chromatography. The flag-tag was used to measure the level of expression. The modified gene was then engineered into the expression vector.

*Expression: Spodoptera frugiperda* (Sf9) cells were grown in a small volume of pre-warmed ES 921 media (Expression Systems, St. Davis, CA, USA). Once the cell density reached 2 × 10^6^ cells/mL, the cells were diluted with pre-warmed ES 921 leucine-depleted media (Expression Systems) to a cell density of 0.6 × 10^6^ cells/mL. The cells were infected with baculovirus at a dilution of 1:2000 when they had reached a cell density of 1 × 10^6^ cells/mL. At about 15 h and 48 h post infection, 150 mg/L and 300 mg/L of ^15^N/^13^C amino acids (Cambridge Isotope Laboratories, Inc., Cambridge, MA, USA) were respectively added to the cells. Once the cells’ diameter had increased 3–4 µm and the cell viability was just below 95%, the cells were centrifuged at 5000× *g* for 10 min at 4 °C. The supernatant was removed and the cell pellets stored at −80 °C. For the Western blot, cell samples of 1 mL were collected every 24 h after infection. The samples were centrifuged at 13,700× *g* for 3 min and stored at −20 °C. 

*Purification:* The frozen cell pellets of the insect cell membranes were dissolved in a buffer containing 50 mM phosphate, 200 mM NaCl, 8 M urea, and 1% DM at pH = 7.4. Extensive vortexing was performed at room temperature and the supernatant was isolated by centrifugation at 4500 rpm for 30 min. The supernatant was incubated with nickel NTA resin (Qiagen, Germantown, MD, USA) for at least 12 h at room temperature. After 12 h, the resin was washed with five column volumes of 50 mM phosphate, 200 mM NaCl, 8 M urea, 1% DM, and 5 mM imidazole at pH = 7.4. After washing, TMH67H8 was eluted from the resin with 50 mM phosphate, 200 mM NaCl, 0.1% DM, and 200 mM imidazole at pH = 7.4. The protein was precipitated from solution by extensive dialysis against 50 mM phosphate pH = 7.4. The precipitated protein was washed several times with 50 mM phosphate buffer and lyophilized in 60% 1,1,1,3,3,3-hexafluoro-2-propanol.

### 2.3. Reconstitution of TMH67H8 and NMR Experiments

The organic solvent samples were prepared by directly dissolving the lyophilized samples in the appropriate organic solvent:water (30% TFE or HFIP) mixture. The reconstitution of TMH67H8 into bicelles was performed by slowly diluting the protein solution in 100 mM DDM into a 10-fold excess of 15% total lipids (DMPC/DHPC, molar ratio = 0.25) (Avanti Polar Lipids, Alabaster, AL, USA) containing 10 mM phosphate and 20 mM KCl, pH = 6.7, at room temperature [13]. The TMH67H8 in the bicelles solution was then centrifuged in a 30 kDa cutoff membrane to a volume of about 400 µL and total protein-to-lipid ratio of 1:50. The detergent micelle samples were prepared by dissolving the appropriate amount of lyophilized TMH67H8 in 100 mM DDM or 100 mM DPC in 10 mM phosphate and 20 mM KCl, pH = 6.7, to give final protein-to-lipid molar ratio of 1:50. All NMR spectra were recorded at 318 K on an Agilent (Varian, Palo Alto, CA, USA) 600 MHz VNMRS spectrometer equipped with a triple-resonance probehead. DSS (4,4-dimethyl-4-silapentane-1-sulfonic acid) was used as chemical shift reference. Two-dimensional ^1^H-^15^N-TROSY spectra were recorded with ranges from 320 to 448 transients per increment, acquisition times of 41 ms (t_1_, ^15^N) and 106 ms (t_2_, ^1^H), and a time domain data size of 80(t_1_) × 1024(t_2_) complex points.

### 2.4. Circular Dichroism Experiments

Small micelles composed of either 1,2-dihexanoyl-*sn*-glycero-3-phosphocholine (D-6-PC,) or DPC were prepared in 10 mM Tris buffer, pH ~7.5, containing 0.5 mM EDTA and 10 Mm NaCl. The hCB2 TMH67H8 peptide was dissolved in 30% TFE/H_2_O or incorporated into micelles at a 100:1 lipid-to-peptide molar ratio. Analysis of peptide secondary structure was then performed with a nitrogen-flushed J-810 spectropolarimeter (Jasco Inc., Easton, MD, USA) controlled by Spectra Manager (version 1.15.00, Jasco Instruments, Easton, MD, USA). Spectra were recorded from 280 to 185 nm at 23 °C using a 1 cm cuvette, a scan rate of 1 nm/min, and an average of three scans per sample.

### 2.5. Molecular Dynamics of TMH67H8

Twelve-hundred-picosecond simulations of TMH67H8 were undertaken here using the Desmond molecular dynamics software package with an OPLS-AA 2005 force field including data for the POPC (Palmitoyl-2-oleoyl-*sn*-glycero-3-phosphocholine) bilayer, along with the simple point charge (SPC) model for water. Force field parameters for TMH67H8 were constructed by utilizing existing parameters for similar atom types. In the simulation setup, M-SHAKE constraints were used on all covalent bonds involving hydrogen atoms to allow an iteration time step of 2 fs. The reference system propagator algorithm (RESPA) was used to integrate the equations of motion using periodic boundary conditions on this orthorhombic simulation system (35 Å × 30 Å × 20 Å). The initial confirmations for TMH67H8 in the POPC bilayer were constructed by placing the minimized solute into pre-equilibrated POPC bilayer (approximate 80 POPC each layer, and 13,240 waters) according to the hypothesis therein. The simulation was stable after 1200 ps. 

## 3. Results

### 3.1. Membrane-Mimetic Chemical Structures

**Figure 1:**
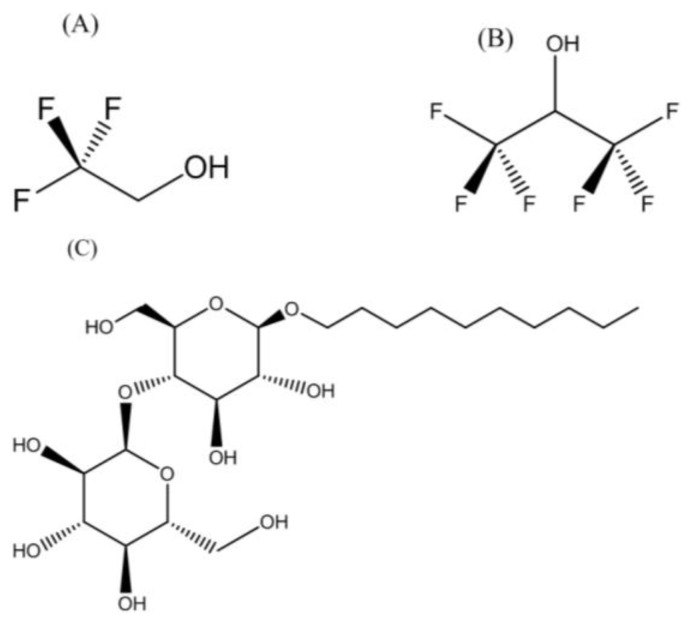
Chemical structures of the molecules used to create the biomimetic environment for reconstituting transmembrane helix, TMH67H8, of the human cannabinoid receptor 2. (**A**) Trifluroethanol; (**B**) hexafluoroisopropanol and (**C**) *n*-decyl-β-d-maltopyranoside.

**Figure 2:**
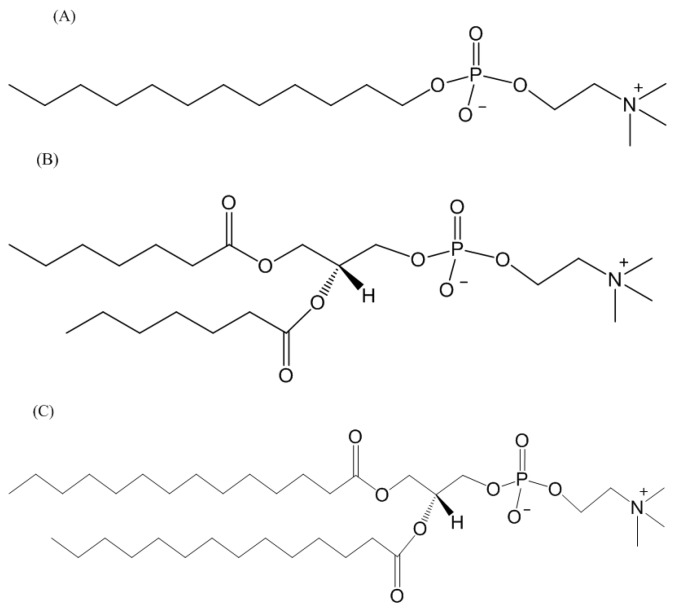
Chemical structures of (**A**) *n*-dodecylphosphocholine; (**B**) 1,2-dihexanoyl-*sn*-glycero-3-phosphocholine, and (**C**) 1,2-dimyristoyl-*sn*-glycero-3-phosphocholine lipids. The 2-dihexanoyl-*sn*-glycero-3-phosphocholine and 1,2-dimyristoyl-*sn*-glycero-3-phosphocholine lipids were mixed in ratios up to 1:4.2 to form the bicelles of different sizes.

The structures of TFE and HFIP are displayed in Figure 1A,B. These alcohols are often used to induce alpha-helical properties on proteins, although the mechanism through which they promote alpha-helical properties is unknown. The structure of the DDM detergent revealed dual hydrophobic (the lipid tail) and hydrophilic (maltose) properties that facilitate lipid displacement and provide a lipid-like environment for membrane proteins (Figure 1C). DPC is a synthetic phosphodiester detergent with the phosphoric acid having ester bonds between choline, (CH_3_)_3_N(CH_2_)_2_OH, and dodecyl alcohol, CH_3_(CH)_11_OH (Figure 2A). Unlike DPC, DHPC and DMPC have two acyl chains, hexanoyl alcohol, (CH_3_(CH_2_)_5_OH, and myristoyl alcohol, (CH_3_(CH_2_)_13_OH, respectively bonded to a glycerol backbone, and both having phosphodiester bond with the phosphoric acid linking to a choline (CH_3_)_3_N(CH_2_)_2_OH group (Figure 2B,C).

### 3.2. Insect Cells Expression, Western Blots Analysis, CD Measurements, and Solution NMR of TMH67H8

**Figure 3:**
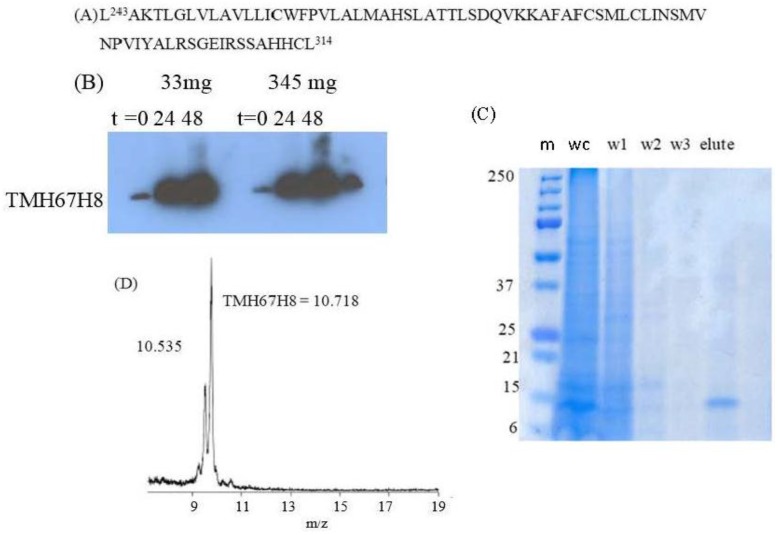
(**A**) The amino acid sequence corresponding to TMH67H8; (**B**) Western blot of CB2-678 expression in 100 mL of *Spodoptera frugiperda* (Sf9) cells in ESF921 leucine-depleted media (Expression Systems). Cells were infected 1:2000 with TMH67H8 when cells were at a density of 0.79 × 10^6^ cells/mL. Leucine was added at *t* = 24 h, 33 mg and at *t* = 48 h, 345 mg, and aliquots were removed to analyze the protein expression as a function of time. Primary antibody, monoclonal anti-Flag M2 (Sigma F1804-1MG, St. Louis, MS, USA), with a dilution of 1:1000 was used. Secondary antibody used was ECL anti-mouse IgG, horseradish peroxidase-linked F(ab′)2 fragment (from sheep) (GE Healthcare, NA9310, Chicago, IL, USA) with a dilution of 1:4000. Marker used was Precision Plus Protein Standards, Kaleidoscope (Bio-Rad, 161-0375, Hercules, CA, USA; (**C**) Coomassie-stained gel of purified TMH67H8, where “m”: marker, “wc”: whole cells, “w1, w2, w3”: wash 1, 2, and 3, respectively, and elute and (**D**) MALDI-TOF analysis of TMH67H8. The peak with molecular weight 10.5 represents THM67H8-methionine.

**Figure 4:**
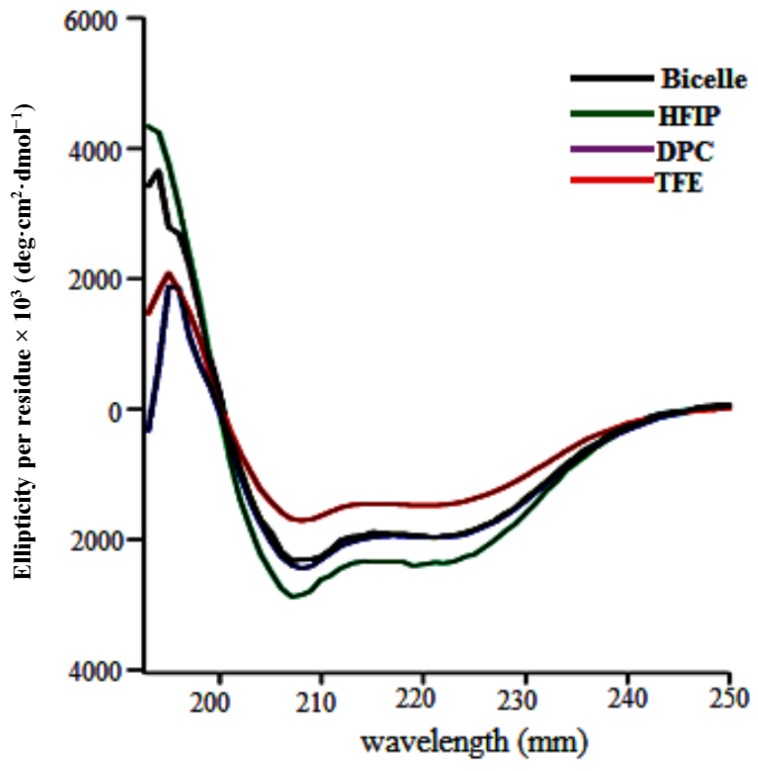
Circular dichroism spectra of TMH67H8 in various membrane mimetic environments.

**Figure 5:**
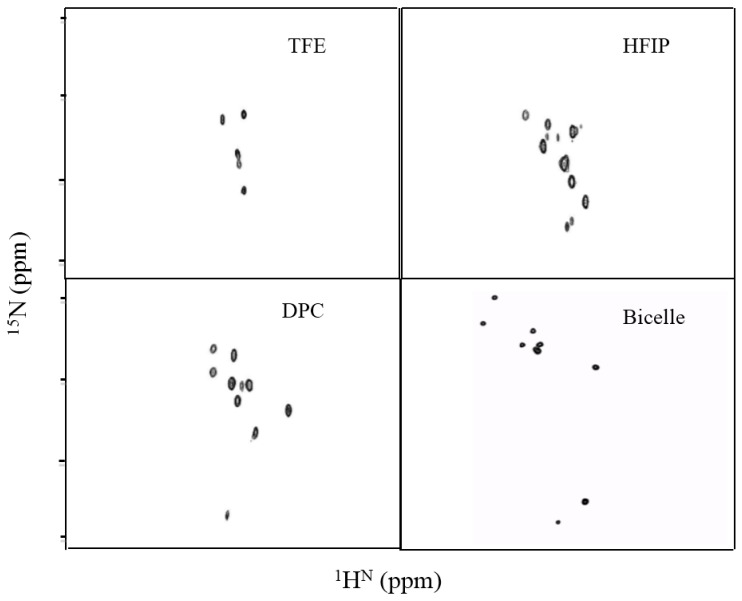
Two-dimensional ^1^H-^15^N HSQC solution NMR spectrum of selectively ^15^N-Leu-labeled TMH67H8 in trifluoroethanol (TFE), 1,1,1,3,3,3-hexafluoro-2-propanol (HFIP), *n*-dodecylphosphocholine (DPC), and dimyristoylphosphocholine/dihexanoylphosphocholine (DMPC/DHPC) isotropic bicelles (*q* = 0.16).

The gene sequence corresponding to TMH67H8 was cloned into the baculovirus expression systems (Figure 3A). The baculovirus-based expression systems are increasingly becoming the most powerful vehicle for producing functional enzymes and receptors [37,38]. Different media conditions were tested to express both selectively and uniformly labeled TMH67H8 in Sf9 insect cells. Western blot analysis utilizing Flag antibody reveals a molecular mass of about 10.6 kDa, as estimated relative to protein markers (Figure 3B). The purification of TMH67H8 was conducted using His-tagged-based purification (Figure 3C) and the purity of the purified protein ascertained by matrix-assisted laser desorption ionization time-of-flight (MALDI-TOF) analysis (Figure 3D). The structural responsiveness of TMH67H8 to the membrane mimetics environments were assessed using circular dichroism (CD) measurements (Figure 4). The shapes and intensities of the representative CD spectra revealed significant alpha-helical structure of the peptide in HFIP and less-structured peptide in TFE membrane-mimetic environments. The CD measurements showed that TMH67H8 folding in the detergent micelles D-6-PC or DPC and the bicelles comprising of DMPC/DHPC (*q* = 0.16) seemed to show no significant differences. TMH67H8 is dominated by clusters of amino acids including leucine (Leu) that are alpha-helix-preference residues and as such can be used to assess the folding pattern of the protein in the various membrane-mimetic environments using solution NMR. We chemically modified all 14 Leu amino acids in TMH67H8 with their ^15^N-Leu isotopic variants to screen the best conditions for expression and reconstituting the selectively labeled receptor domain. As shown in Figure 5, ^1^H-^15^N TROSY experiments were conducted in four different environments including two alcohols (TFE and HFIP), one detergent micelle (DPC), and isotropic bicelles (DMPC/DHPC, *q* = 0.16). In TFE, 5 out of the 14 Leu amino acids were identified, whereas 12 in HFIP for the same protein concentration and organic/water ratio (Figure 5). Ten out of the 14 cross peaks were identified in DPC detergent micelles (Figure 5). In light of the current understanding of bicelle properties as bilayer mimetics and the ability of these bicelles to support the folding and stability of membrane proteins, ^1^H-^15^N TROSY-heteronuclear single quantum coherence or correlation (HSQC) experiments were also conducted in isotropic bicelles of *q* = 0.16 (Figure 5). As indicated, the spectrum displayed well-resolved 9 homogenous cross peaks, indicating proper folding of the receptor domain in the isotropic bicelles microenvironment.

### 3.3. NMR Analysis of TMH67H8 as a Function of Bicelle q Ratio

**Figure 6:**
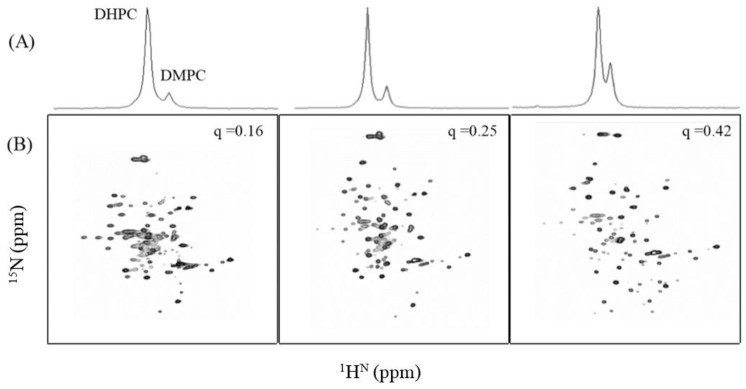
(**A**) ^31^P NMR spectra of TMH67H8 in isotropic phospholipid bicelles at 35 °C. (**A**) DMPC:DHPC, *q* = 0.16, 0.25, and 0.42, in that order. The ^31^P signals from the DHPC and DMPC are indicated; (**B**) two-dimensional ^1^H-^15^N HSQC solution NMR spectrum of TMH67H8 in the respective DMPC:DHPC isotropic bicelles.

While the size of the TFE, HFIP, and DPC cannot be varied upon changes in concentration, isotropic bicelle size can be influenced by adjusting the concentrations of the longer chain DMPC lipids. As shown in Figure 6A, ^31^P NMR was used to estimate the DMPC/DHPC ratio by titrating DMPC into the bicelles to obtain *q* ratios of 0.16, 0.25, and 0.42. The corresponding ^1^H-^15^N TROSY-HSQC experiments were conducted on uniformly ^15^N-labeled TMH67H8 in all three molar *q* ratios and the folding of the receptor domain investigated (Figure 6B). It is evident that by increasing the concentration of DMPC in the bicelle system, the number of cross peaks and the extent of dispersion of the resonances in the ^1^H-^15^N TROSY spectra improve (from *q* = 0.16 to *q* = 0.42). It was also observed that protein precipitation during the NMR experiments decreases as we move from low DMPC concentration (*q* = 0.16) to high (*q* = 0.42). This observation demonstrates that the size of the membrane is crucial in stabilizing and maintaining the proper conformation of the receptor domain.

### 3.4. Modeling TMH67H8 in a Membrane-Mimetic Environment

**Figure 7:**
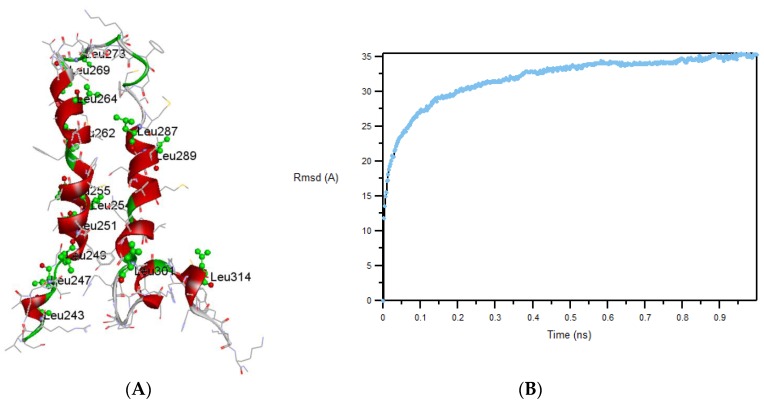
(**A**) Molecular dynamics (MD) simulation of TMH67H8 in a model membrane of DMPC and (**B**) root-mean-square-deviation (RMSD) profile for 900 ps simulation time.

TMH67H8 was modeled in a POPC environment to investigate the interaction and orientation of the receptor fragment. Figure 7A shows the structure of TMH67H8 after 900 ps of simulation. Clearly, Leu269, Leu273, and Leu287 are located in the unstructured extracellular loop 3 (EC3) region of TMH67H8, whereas L314 is located in the intracellular helix 8 portion of the full receptor. The cartoon conforms to the NMR structure of TMH67H8 that has already been published by Tiburu et al. [21]. The energy-minimized root-mean-square-deviation (RMSD) profile is displayed in Figure 7B after 1200 ps of simulation.

## 4. Discussion

TFE and HFIP media have been used in the past to determine the structure of similar transmembrane proteins and receptor fragments because of their ability to stabilize their alpha-helical structure [14,28,39,40,41]. The secondary structures stabilized by either TFE and HFIP were often assumed to reflect conformations that prevail in the early stages of protein folding [42,43]. We therefore tested the ability of these organic solvents to solubilize and stabilize TMH67H8 conformation for NMR studies. The most noticeable difference between the TFE and HFIP NMR spectra was that fewer cross peaks were identified in the former, suggesting TFE is the least helix-inducing organic solvent for TMH67H8 conformation. HFIP, an alcohol with six fluorine atoms, had demonstrated to be the most effective membrane-mimetic environment. It was believed that the presence of two CF3 groups made HFIP a better H-bond donor, resulting in a more compact TMH67H8 structure as observed in other NMR studies of membrane proteins in HFIP [28]. To further validate the suitability of using HFIP for structural characterization of the receptor domain, we performed similar experiments with uniformly ^15^N-labeled TMH67H8. The ^15^N HSQC spectrum produced in HFIP was broad with very few resolved peaks (data not shown). The results indicated that the spread of the HSQC resonance peaks in HFIP did not reflect a properly folded TMH67H8 and, thus, the receptor conformational integrity was not maintained, even though the selectively labeled receptor showed promise in its utilization for structural studies.

Most detergent micelles, including DPC, have also been utilized in solubilizing membrane proteins for both X-ray and solution NMR studies [10,44]. DPC in particular contains spatially distinct hydrophobic and hydrophilic regions that can influence the structural integrity of TMH67H8 and can affect the overall shape and distribution of the HSQC resonance peaks. Thus, DPC might be another important solubilizing agent for TMH67H8 due to its ability to better protect the TM region of the receptor domain as a result of protein–lipid interactions that match the hydrophobic length of DPC to the alpha-helical regions of TMH67H8. The cross peaks in the DPC spectrum of ^15^N selectively labeled TMH67H8 were sharp and more dispersed compared to the HFIP spectrum. Again, we tested the feasibility of using DPC for structural determination of uniformly labeled TMH67H8, but as in HFIP, DPC failed to produce well-dispersed cross peaks (data not shown). When different concentrations of DPC were used to reconstitute the receptor domain, more heterogeneous and disproportionally varied resonance peaks were produced that indicated there were secondary structural fluctuations within TMH67H8 in the DPC environment regardless of the concentration of the detergent micelle.

Our next option was to reconstitute the receptor domain bicelles comprising a mixture of DMPC and DHPC. The complex was investigated using ^1^H-^15^N TROSY-HSQC resonances to observe the extent of folding of TMH67H8. There was clear evidence that DMPC/DHPC *q* = 0.25–0.42 provided well distinct resonance peaks suggesting the existence of a critical window of DMPC/DHPC *q* ratio can support the conformation of membrane proteins for structural determination. This *q* ratio window seemed to be transmembrane (TM) protein dependent and one TM protein that prefers a particular *q* ratio may not necessarily apply to another TM protein. These findings will allow further development of novel membrane mimetics to provide greater diversity of lipid mixtures than those currently being employed for GPCR stability and folding, which is critical for both X-ray and NMR studies of GPCRs.

## Abbreviations

The following abbreviations are used in this manuscript:

DHPC	Dihexanoylphosphocholine
DMPC	Dimyristoylphosphocholine
DDM	*n*-Dodecyl-β-d-Maltopyranoside
DPC	*n*-Dodecylphosphocholine
DSS	4,4-dimethyl-4-silapentane-1-sulfonic acid
GPCRs	G-protein coupled receptors
hCB2	human cannabinoid receptor 2
HSQC	heteronuclear single quantum coherence or correlation
Leu	Leucine
MALDI-TOF	Matrix-assisted laser desorption ionization time-of-flight
TFE	Trifluoroethanol
HFIP	1,1,1,3,3,3-hexafluoro-2-propanol
RMSD	Root-mean-square-deviation
Sf9	Spodoptera frugiperda
TMH	Transmembrane helix
TROSY	Transverse relaxation-optimized spectroscopy
